# Design of Two-Dimensional Transient Circular Thermal Cloaks with Imperfect Interfaces

**DOI:** 10.3390/ma16062297

**Published:** 2023-03-13

**Authors:** Jun-Hong Lin, Tungyang Chen

**Affiliations:** 1 Department of Civil Engineering, Chung Yuan Christian University, Zhongli Dist., Taoyuan City 320314, Taiwan; jhlin@cycu.edu.tw; 2 Department of Civil Engineering, National Cheng Kung University, Tainan 70101, Taiwan

**Keywords:** thermal metamaterials, transient response, imperfect interfaces, thermal invisibility, scattering cancellation

## Abstract

In this paper, analytic modeling for the design of a transient thermal invisibility cloak with imperfect interfaces is presented together with numerical simulations. In contrast to steady-state conditions, it is shown that an object can only be made partially invisible under a transient-state condition with either ideal or imperfect interfaces. The thermal visibility of an object to the external region can be optimally suppressed under certain conditions referred to as the “weak invisibility conditions” for the transient response, which are different from the “strong invisibility conditions” that can completely conceal an object in a steady state. In the formulation, a homogeneous metamaterial with constant volumetric heat capacity and constant anisotropic conductivity tensor is employed. It can be demonstrated that the interface’s bonding conditions will have a significant effect on the design of metamaterials. Two typical types of imperfect interfaces, referred to as low-conductivity- and high-conductivity-type interfaces, are considered. Conditions, that render an object mostly undetectable, are analytically found and expressed in simple forms under quasi-static approximations. Within the quasi-static limit, the thermal localization in the target region can be tuned with the anisotropy of the conductivity tensor. Thermal shielding or concentrating effects in the target region are exemplified based on finite element simulations to demonstrate the manipulation of heat flux in the target region. The present findings make new advances in theoretical fundamentals and numerical simulations on the effect of the imperfect interface in the transient regime and can serve as guidelines in the design of thermal metamaterials through the entire conduction process.

## 1. Introduction

Inspired by the novel innovation of an electromagnetic invisibility cloak proposed by Pendry et al. [1], thermal invisibility cloaks with tunable functionalities have attracted substantial attention in recent years [2,3,4,5,6,7,8,9,10,11,12,13] for various applications in advanced thermal management. Thermal metamaterials are often composed of different materials with internal structures. The bonding conditions between different constituent materials will in general affect the heat-transferring behavior across the interface, causing diminishing or even breaking down the functionalities of the metamaterials. For example, Zheng and Li [14] demonstrated that, in the presence of interfacial thermal resistance, the temperature field outside the cloak could be greatly distorted, if the interfaces are inappropriately considered to be perfect. In practical situations, imperfectness always exists across the interface between two different solids in contact owing to the interfacial thermal resistance [15] or roughness at their common boundaries, or caused by the mismatch of physical properties between the adjacent phases [14]. The effect of imperfect interfaces will become particularly significant and non-negligible while the thermal device is in the nano-scaled [15,16]. However, most previous studies mainly considered ideal interfaces including steady-state and transient state response, see for example [2,3,4,5,6,7,8,9,10,11,12,13,17,18,19,20,21,22,23,24,25,26] and [13,14,15,16,17,18,19,20,21,22]. A recent progress in the study of the imperfect interface effect in the design of thermal metamaterials was made by Chen and Lin [27,28] for conduction phenomena in the steady-state regime. In these studies, they showed that an object can always be made thermally invisible, with the effect of the imperfect interface, as that of a homogeneous background material. This invisibility condition was analytically derived in simple and well-structured expressions. In addition, the effect of material and geometric parameters on thermal shielding and enhancements are exactly identified.

This present work is an endeavor along this line of research with a focus now on the transient state, that is the design of transient thermal metamaterials with imperfect interfaces effect. Two different types of imperfect interfaces, low conductivity—(LC) and high conductivity- (HC) types, are considered. The LC-type interface exhibits an interfacial thermal resistance [15], where the normal component of heat flux is continuous and the temperature will have a jump across the interface, characterized by a scalar interface parameter β. On the other hand, an HC-type interface [29] is physically opposite to that of an LC-type interface. An interface of HC-type is that across the interface the temperature will remain continuous, whereas a discontinuous jump in normal heat flux can be described by a specific interface parameter α. A detailed exposition of interface conditions from the mathematical framework was introduced by Benveniste and Miloh [30]. In the present study, we consider a homogeneous metamaterial, with constant anisotropic conductivities and volumetric heat capacity. Our objective is to examine the conditions under which the exterior thermal field will not be disturbed with the introduction of the metamaterial layer, and that to examine the extent of thermal localization in the target region. The effect of imperfect interfaces are examined analytically, to capture the underlying physical implications. In contrast to the cases of steady-state conduction [27], it can be found that, on a macroscale, governed by Fourier’s law with transient effects, an object can only be made partially invisible irrespective of bonding conditions. The disturbance in the background host medium can be minimized by eliminating finite primary modes of scattered fields based on the concept of the scattering cancellation technique (SCT) [31]. This can be referred to as the “weak invisibility conditions”, analogous to the terminology adopted in [32]. For a steady state condition, however, it is possible to completely conceal an object perfectly [27], which it was referred to as a “strong invisibility condition”. Within the target region, we find that the manipulation of heat flux, which is perturbed over time in the transient state, remains controlled by the anisotropy ratio of the designed metamaterial within a quasi-static limit. The perturbation of the heat flux in the core region will decay with time. This suggests that a stable state of thermal localization, which will be the same as that described in [27], can be achieved as the steady-state field. On the other hand, the magnitude of volumetric heat capacity will govern how long it will take to lead to converged steady-state fields. While still complying with the framework of laws of thermodynamics, these universal connections, together with the strong invisibility condition reported in [27], offer a guideline for the design of thermal metamaterials through thermal conduction process for both transient and steady responses with the effect of imperfect interfaces. The analytic results will be presented in detail. The manipulation of the thermal localization in the core region will be illustrated together with numerical simulations based on finite element simulations. In addition, extending from our previous framework [27], a strategy for the design of a multi-layered thermal cloak systematically through a series of homogenization procedures will be proposed and demonstrated with numerical simulations.

Relevant works on thermal metamaterials with transient effects, but with ideal interfaces, can be found in a few studies. Among them, Xu et al. [23] reported that a three-dimensional cloak made of an ultrathin layer of isotropic material can act as an excellent invisibility cloak regardless of the volumetric heat capacity if the thickness of the cloak is negligibly small. Farhat et al. [24] showed that a spherical cloak made of homogeneous and isotropic material can efficiently suppress the scattered heat around the thermal cloak in the transient regime as if the object is nearly not present. Sklan et al. [25] found that a thermal cloak made of inhomogeneous conductivity and constant volumetric heat capacity, which works perfectly in the steady state, is always detectable with a small amount of scattered heat in the transient state. Ji et al. [26] explored the cloaking of a complex shape on the basis of either the concept of neutral inclusion or the transformation method in both steady-state and transient regimes and showed that the neutral inclusion method is more flexible and easier in practical implementation. Zhang et al. [33] reported that the transient heat flow velocity can be controlled with the regulation of effective density and specific heat capacity via a proposed thermal architected metamaterial. To our knowledge, the subject of the present context is new in the literature.

## 2. Results

Based on the first principle of thermodynamics, the temperature field T is governed by Fourier’s relation $\nabla \cdot k\nabla T=\rho c_{p}\left(\partial T/\partial t\right)$ in the absence of a heat source, in which k is the conductivity tensor, ρ the mass density, and $c_{p}$ the specific heat capacity at constant pressure. The total response of the temperature field consists of the transient and the steady-state responses. The former will decay with time while the latter governs the long-term response. Apart from the steady-state response, the volumetric heat capacity $s=\rho c_{p}$ plays an essential role in a transient response during the heat conduction process. When the volumetric heat capacity s or ∂T/∂t is very small, the state change of the system will be negligibly small and thus the heat conduction process will asymptotically proceed under the “quasi-static process”. Incorporating the effect of imperfect interfaces, here we will extend the framework from our previous work [27] to explore how a thermal cloak can be properly designed in the transient regime under the quasi-static approach.

**Figure 1:**
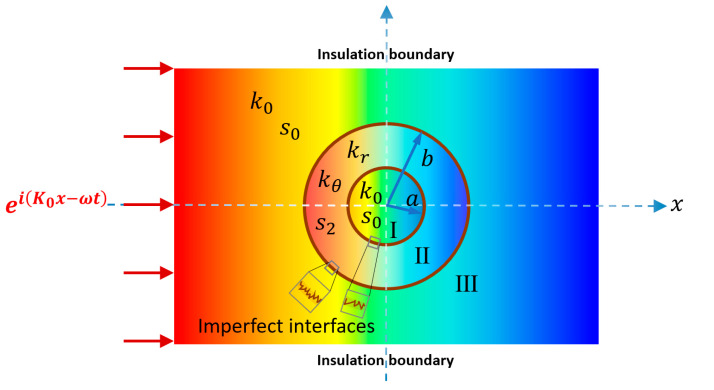
Schematic illustration of a cylindrical thermal device under a transient-state condition. The interfaces at r=a and r=b are imperfectly bonded. A harmonic temperature excitation $e^{i\left(K_{0}x-\omega t\right)}$ is applied on the left-hand side, where ω is the angular frequency, and $K_{0}=\sqrt{i\omega s_{0}/k_{0}}$ the wave number. Region I and Region III are made of the same isotropic material with constant conductivity $k_{0}$ and constant volumetric heat capacity $s_{0}$. Region II is the designed anisotropic metamaterial with constant volumetric heat capacity $s_{2}$, constant conductivities $k_{r}$ and $k_{\theta }$.

A two-dimensional model is illustrated in Figure 1. Region I is the target region for heat flux control, made of the same material as the surrounding host medium Region III with constant isotropic conductivity $k_{0}$ and volumetric heat capacity $s_{0}$. Region II is the designed metamaterial with constant volumetric heat capacity $s_{2}$, and constant anisotropic conductivity tensor, expressed as

(1) $$k=\left(\begin{array}{cc} k_{r} & 0\\ 0 & k_{\theta } \end{array}\right),$$

where $k_{r}$ and $k_{\theta }$ are respectively the constant radial and circumferential conductivities. Both of the interfaces at r=a and r=b are considered as either LC- or HC-type imperfect interfaces. It is mentioned that, by eliminating the effect of imperfect interfaces, the analytic result for the case of perfect interfaces will be readily obtained. The transient thermal excitation $T_{inc}=U_{inc}e^{-i\omega t}=e^{i\left(K_{0}x-\omega t\right)}$ is applied horizontally along x axis from the left-hand side of the thermal device, where $K_{0}=\sqrt{i\omega s_{0}/k_{0}}$ refers to the wave number of the pseudo diffusion plane wave in Region III. The scalar temperature field $U_{inc}$ can be expanded in cylindrical coordinates by the Jacobi-Anger expansion [34], given as

(2) $$U_{inc}\left(r,\theta \right)=e^{iK_{0}x}=e^{iK_{0}r\cos\theta }=\sum_{n=-\infty }^{\infty }i^{n}J_{n}\left(K_{0}r\right)e^{in\theta },$$

in which $J_{n}$ refers to the Bessel function of the first kind. Taking the boundary conditions and geometric symmetry into account, the temperature field for the transient response can be assumed as $T\left(r,\theta ,t\right)=U\left(r,\theta \right)e^{-i\omega t}$. As such, the scalar temperature fields that comply with Fourier’s relation can be expressed in cylindrical coordinates as [34]

(3) $$\begin{array}{c} U_{1}\left(r,\theta \right)=\sum_{n=-\infty }^{\infty }A_{n}J_{n}\left(K_{0}r\right)e^{in\theta },\;r\leq a,\\ U_{2}\left(r,\theta \right)=\sum_{n=-\infty }^{\infty }\left(B_{n}J_{\lambda_{n}}\left(K_{2}r\right)+C_{n}H_{\lambda_{n}}^{\left(1\right)}\left(K_{2}r\right)\right)e^{in\theta },\;a<r\leq b,\\ U_{3}\left(r,\theta \right)=\sum_{n=-\infty }^{\infty }i^{n}\left(J_{n}\left(K_{0}r\right)+S_{n}H_{n}^{\left(1\right)}\left(K_{0}r\right)\right)e^{in\theta },\;r>b, \end{array}$$

where $H_{n}^{\left(1\right)}$ is Hankel function of the first kind with order n, $K_{2}=\sqrt{i\omega s_{2}/k_{r}}$ is the wave number in Region II, and the $n^{th}$-order anisotropy parameter is equal to $\lambda =\sqrt{k_{\theta }/k_{r}}$ multiplied by n, given as $\lambda_{n}=n\sqrt{k_{\theta }/k_{r}}=n\lambda$. The value of λ represents the anisotropy of the conductivities in Region II. A value of λ=1 stands for an isotropic material in Region II. The coefficients $A_{n}$, $B_{n}$, $C_{n}$ and $S_{n}$ will be determined by the interface conditions at the interfaces. To make the thermal cloak undetectable, the scattering coefficients $S_{n}$ have to be canceled out. However, for the transient response, it is not possible of eliminating the infinite scattering modes $S_{n}$ by a limited number of boundary conditions and interface conditions. As such, one can only get rid of the primary modes of scattering fields to suppress the entire scattering fields to the maximum extent such that the thermal cloak can be made mostly invisible. The scattering width $\sigma_{2D}$, which is introduced to measure the overall visibility of the inclusion to external observers, can be defined as [35]

(4) $$\sigma_{2D}=\underset{r\to \infty }{\lim}\int \frac{{\left|T_{sc}\right|}^{2}}{{\left|T_{inc}\right|}^{2}}d\Omega ,$$

where $T_{sc}=T_{3}-T_{inc}$ denotes the scattering fields in Region III. The subscript 2D refers to the two-dimensional or cylindrical cases. With the substitution of (3), it will be shown that

(5) $$\sigma_{2D}=\frac{4}{\left|K_{0}\right|}\sum_{n=-\infty }^{\infty }{\left|S_{n}\right|}^{2}.$$

The detailed derivation of (5) is given in Appendix A. It is remarkable that in the quasi-static approximation, ∂T/∂t=−iωT shall be very small. This requires a very small frequency ω→0 as well as a long diffusion length in each region, namely $K_{0}r\ll 1$ in Region I and $K_{2}r\ll 1$ in Region II. In this scenario, the scattering fields are mostly contributed by the primary scattering orders, i.e., the first two orders (n=0 for the monopole and n=1 for the dipole mode). Consequently, the scattering width will be asymptotically equal to

(6) $$\sigma_{2D}\approx \frac{4}{K_{0}}\left({\left|S_{0}\right|}^{2}+{\left|S_{1}\right|}^{2}\right).$$

It turns out that, on the basis of the concept of SCT [31], the scattering fields can be mostly suppressed when $S_{0}$ and $S_{1}$ are zero. This will give the “weak invisibility conditions” for the transient thermal cloak that can mostly conceal the inclusion. It is remarkable to note that this also coincides with the results for the spherical case that the perturbation around the thermal cloak is primarily governed by the first two modes of the scattered field as well in the quasi-static limit, as illustrated in [24]. We mention that the thermal cloak that fulfills weak invisibility physically implies that the effective material parameters of the thermal device are equivalent to those of the background medium in an averaging sense [32]. This concept also corresponds to that proposed by [36] for determining the effective shear modulus of a composite material based on a generalized self-consistent method. In the following, these exact connections for different types of bonding conditions will be derived in a simple form. In the following the universal connections as the criteria for the design of homogeneous metamaterial to optimally achieve thermal invisibility will be analytically examined respectively for the cases of ideal interfaces, LC- and HC-type interfaces in Section 2.1, Section 2.2 and Section 2.3. The main results will be given in Equations (7) and (8) for ideal interfaces, Equations (10) and (11) for LC-interfaces, and Equations (15) and (16) for HC-interfaces. 

### 2.1. Ideal Interface

The case of ideal interfaces will be examined first. In a steady state, the design of a thermal invisibility cloak only involves the connection that balances the equivalence in effective conductivities between the surrounding background material and the cloaked inclusion. Additionally, in the transient regime, the volumetric heat capacity will take effect on the dynamic balance as well. In the quasi-static limit, it is analytically found that when the universal connections

(7) $$s_{2}=s_{0},\;\mathrm{for}\;S_{0}=0\;(\mathrm{monopole}\;\mathrm{mode}),$$

(8) $$k_{0}=\sqrt{k_{r}k_{\theta }},\;\mathrm{for}\;S_{1}=0\;(\mathrm{dipole}\;\mathrm{mode}),$$

are fulfilled, the cloak can be made least visible to the exterior observers. It is of interest that these two constraint conditions confine separately the balance in volumetric heat capacities and conductivities. In the quasi-static limit, we find that the volumetric heat capacity is necessary to be homogeneous throughout based on the monopole constraint (7), whereas the dipole mode (8) gives an identical connection to the invisibility condition for the steady-state response [37]. This implies that the design of thermal invisibility for the transient response will work perfectly in a steady state. As will be mentioned later, depending on whether the interfaces are of LC- or HC-type, the effective conductivities of the cloaked region will be diminished or enhanced. To signify the effect of the imperfectness at the interfaces, let us define an imperfectness parameter $g=k_{0}/k_{G}=k_{0}/(\lambda k_{r})$. Note that $k_{G}=\lambda k_{r}=\sqrt{k_{r}k_{\theta }}$ refers to the geometric mean of the conductivities of the metamaterial in Region II. Thus (8) can be simplified as g=1. This also indicates that, in the case of ideal interfaces, the connections for thermal invisibility only depend on material properties $s_{2}$ and $k_{G}$ for the metamaterial, regardless of geometric size or the anisotropy ratio. When the imperfectness of the interfaces starts to increase, the value of g will begin to deviate from unity to achieve thermal invisibility. A value of g<1 and g>1 will be invoked for the cases of LC- and HC-type interfaces to compensate for the reduction or enhancement of effective conductivities respectively. The size effect can be observed when g≠1.

### 2.2. Low Conductivity-Type Interface

The LC-type interface exhibits thermal contact resistance impeding heat flowing across the interfaces, also known as Kapitza resistance [15]. It is mentioned that the interface conditions remain the same for steady and transient states for LC-type interfaces [38]. Specifically, the interface conditions for LC-type interfaces at r=a and r=b can be expressed as [39,40]

(9) $$k_{0}\frac{\partial T_{1}}{\partial n}=k_{r}\frac{\partial T_{2}}{\partial n}={\beta_{a}\left.\left(T_{2}-T_{1}\right)\right|}_{r=a},\;k_{0}\frac{\partial T_{3}}{\partial n}-k_{r}\frac{\partial T_{2}}{\partial n}={\left.\beta_{b}\left(T_{3}-T_{2}\right)\right|}_{r=b}$$

here $T_{1}$, $T_{2}$, and $T_{3}$ are temperature fields in Region I, II, and III, respectively, and ∂/∂n denotes the normal derivative at interfaces. The interface parameter β is defined as the ratio between the temperature jump and the normal heat flux across the interface, with a dimension of W⋅m^−2^K^−1^. The subscripts a and b in β refer to the interfaces at r=a and r=b respectively. A value of β→∞ represents an ideal interface, while in contrast, the case that β→0 stands for the adiabatic contact. With the substitution of (3) into (9), the weak invisibility conditions for the LC-type can be obtained by eliminating $S_{0}$ and $S_{1}$ simultaneously, given as

(10) $$s_{2}=s_{0},\;\mathrm{for}\;S_{0}=0\;(\mathrm{monopole}\;\mathrm{mode}),$$

(11) $$g\frac{\left(g+\left(1+{\tilde{\beta }}_{a}\right)\right)-c^{\lambda }\left(g-\left(1+{\tilde{\beta }}_{a}\right)\right)}{\left(g+\left(1+{\tilde{\beta }}_{a}\right)\right)+c^{\lambda }\left(g-\left(1+{\tilde{\beta }}_{a}\right)\right)}+{\tilde{\beta }}_{b}=1,\;\mathrm{for}\;g<1,\;S_{1}=0,(\mathrm{dipole}\;\mathrm{mode}),$$

where $c={\left(a/b\right)}^{2}$ is the area fraction of Region I. The non-dimensional interface parameters ${\tilde{\beta }}_{a}=k_{0}/\left(a\beta_{a}\right)$ and ${\tilde{\beta }}_{b}=k_{0}/\left(b\beta_{b}\right)$ are defined the same as those in Reference [27] for consistency. The detailed derivation is given in Appendix B. Regarding the effect of imperfectness at the interfaces, it is remarkable to note that, when $\beta_{a}\to \infty$ and $\beta_{b}\to \infty$, that is ${\tilde{\beta }}_{a}={\tilde{\beta }}_{b}=0$, the case of LC-type interfaces will reduce back to that of ideal interfaces. When the values of ${\tilde{\beta }}_{a}$ and ${\tilde{\beta }}_{b}$ increase as the thermal resistance grows at the interfaces, the effective conductivities of the metamaterial have to increase correspondingly to compensate for the effect of impedance across the interfaces. As such, the value of $k_{G}=\sqrt{k_{r}k_{\theta }}$ shall be larger than the conductivity in the background medium $k_{0}$, which leads to $g=k_{0}/k_{G}<1$ for the LC-type cases. Unlike the perfect bonding cases, the weak thermal invisibility relates not only to the material properties $s_{2}$ and $k_{G}$, but also the imperfectness parameter g, area fraction $c={\left(a/b\right)}^{2}$, and $\lambda =\sqrt{k_{\theta }/k_{r}}$. In addition, the LC-type parameters ${\tilde{\beta }}_{a}=k_{0}/\left(a\beta_{a}\right)$ and ${\tilde{\beta }}_{b}=k_{0}/\left(b\beta_{b}\right)$ exhibit size effect for the design of a thermal cloak. The effect of thermal resistance on the thermal cloak will become more significant with scaling down the thermal device. For a detailed exposition of the effect of interfaces, one can also refer to our previous work [27]. In addition, under the quasi-static approximation, we can find these two universal conditions are decoupled, namely, the monopole connection (10) constrains the volumetric heat capacity, while the dipole mode (11) rules the conductivities in Region II separately. We mention that the monopole and dipole constraints will be coupled, related to both volumetric heat capacities and conductivities simultaneously if the process of heat conduction does not proceed slowly enough to fulfill the quasi-static assumption. Only when the rate of temperature change is relatively small during the conduction process will the constraint conditions for these two modes be decoupled. In addition, it can be seen that (11) is exactly the same with the strong invisibility condition for a steady-state response for the case of LC-type interfaces as shown in Reference [27]. These results indicate that, in the quasi-static limit, the system is nearly in static equilibrium at each instant of time during the heat diffusion process. In this scenario, the dipole mode solely connects the steady-state behavior while the time effect of the transient response reflects entirely on the monopole constraint. When the volumetric heat capacity is negligibly small, the dynamic response will asymptotically approach the steady-state response. In this case, one can observe that the monopole constraint (10) becomes trivial, whereas the dipole mode alone governs the universal connection for invisibility entirely. Physically this implies that the inclusion, which can be least revealed with the design via the connections (10) and (11) in a transient state, will be completely concealed as a steady state is established after a certain period of time. This phenomenon also agrees well with the numerical results to be discussed later in Section 5.

Additionally, it is worthwhile to mention that, if the volumetric heat capacity in Region I, denoted as $s_{1}$, is considered different from that in Region III, the monopole condition will become

(12) $$\left(b^{2}-a^{2}\right)s_{2}-b^{2}s_{0}+a^{2}s_{1}=0,\;\mathrm{for}\;S_{0}=0$$

The above equation can be rewritten as

(13) $$\frac{s_{2}-s_{0}}{s_{2}-s_{1}}=c,\;\mathrm{for}\;s_{1}\neq s_{0},S_{0}=0$$

This result is analogous to that found in Reference [24] for a three-dimensional problem. When $s_{1}=s_{0}$, (12) will give c=1 or $s_{2}=s_{0}$. The former is a trivial result indicating that the whole medium is homogeneous throughout. To our concern, the latter $s_{2}=s_{0}$ is selected as the constraint condition for monopole mode, as shown in (10).

### 2.3. High Conductivity-Type Interface

The HC-type interface, in contrast to the LC-type interface, can be modeled in terms of a highly conducting interphase layer [30,41]. The interface conditions of an HC-type interface are derived based on the dynamic balance within the interphase layer with an asymptotic approach. The volumetric heat capacity of the interphase $s_{int}$ have to be taken into account to balance the discrepancy resulting from the discontinuous drop of heat flux in a transient state. A detailed exposition of the derivation of the HC-type interface condition with transient effect is given in Appendix C. The HC-type interface conditions at the interfaces can be expressed as [27]

(14) $${\left.\left(T_{2}-T_{1}\right)\right|}_{r=a}=0,{\left.\left(k_{r}\frac{\partial T_{2}}{\partial r}-k_{0}\frac{\partial T_{1}}{\partial r}\right)\right|}_{r=a}={\left.\left(-\alpha_{a}{∆}_{s}T+s_{s}\frac{\partial T}{\partial t}\right)\right|}_{r=a},\;{\left.\left(T_{3}-T_{2}\right)\right|}_{r=b}=0,{\left.\left(k_{0}\frac{\partial T_{3}}{\partial r}-k_{r}\frac{\partial T_{2}}{\partial r}\right)\right|}_{r=b}={\left.\left(-\alpha_{b}{∆}_{s}T+s_{s}\frac{\partial T}{\partial t}\right)\right|}_{r=b},$$

where the surface volumetric heat capacity of the interphase is defined as $s_{s}=s_{int}h$, and h the thickness of the interphase. Nevertheless, $s_{s}$ is negligible as $s_{int}$ is finite and h→0. The HC-type interface conditions in the transient regime can be reduced back to that in a steady state [29]. The parameters of the HC-type interfaces are represented by $\alpha_{a}$ and $\alpha_{b}$ respectively for the interface at r=a and at r=b, with a dimension of W⋅K ^−1^. In the quasi-static limit, we find that, when the material parameters and geometric parameters fulfill the universal connections

(15) $$s_{2}=s_{0},\;\;\;\;\;\;\;\;\mathrm{for}\;S_{0}=0\;(\mathrm{monopole}\;\mathrm{mode})$$

(16) $$g^{-1}\frac{\left(g\left(1+{\hat{\alpha }}_{a}\right)+1\right)+c^{\lambda }\left(g\left(1+{\hat{\alpha }}_{a}\right)-1\right)}{\left(g\left(1+{\hat{\alpha }}_{a}\right)+1\right)-c^{\lambda }\left(g\left(1+{\hat{\alpha }}_{a}\right)-1\right)}+{\hat{\alpha }}_{b}=1,\;\;\mathrm{for}\;g>1,\;S_{1}=0\;(\mathrm{dipole}\;\mathrm{mode})$$

the visibility to the exterior observers can be minimized. As the transient effect diminishes and only the steady-state response remains, the condition for thermal invisibility will solely rely on the dipole mode constraint (16), which is indeed the strong invisibility condition as introduced in [27]. Again, it is noted that both monopole and dipole conditions will depend on volumetric heat capacities and conductivities simultaneously if the quasi-static approximation, namely $K_{0}r\ll 1$ in Region I and $K_{2}r\ll 1$ in Region II, is not applicable. In (16), the dimensionless parameters ${\hat{\alpha }}_{a}=\alpha_{a}/(ak_{0})$, ${\hat{\alpha }}_{b}=\alpha_{b}/(bk_{0})$ are defined to indicate the bonding situations for the HC-type interfaces. It will give ideal interfaces when $\alpha_{a}\to 0$, $\alpha_{b}\to 0$, namely ${\hat{\alpha }}_{a}\to 0$ and ${\hat{\alpha }}_{b}\to 0$. The connections for weak thermal invisibility will reduce to that of ideal interfaces, which leads to g=1 for the dipole mode condition. The value of ${\hat{\alpha }}_{a,b}$ will increase accordingly with the increase of g, which implies that $k_{G}$ have to be lowered down to balance the high conducting interfaces to achieve thermal invisibility. The case that α→∞ represents the limiting situation that the interfaces are infinitely superconducting [27,42]. Together with the LC- and HC-type interfaces, the relation between the interface parameters ${\tilde{\beta }}_{a}$ and ${\hat{\alpha }}_{a}$ versus g can be illustrated in Figure 2 in [27]. Similar to the case of LC-type interfaces, the thermal invisibility for the case of HC-type interfaces depends on volumetric heat capacity $s_{Ⅱ}$, as well as g, c, and λ. One can observe that ${\hat{\alpha }}_{a}=\alpha_{a}/(ak_{0})$ and ${\hat{\alpha }}_{b}=\alpha_{b}/(bk_{0})$ will become noticeable in magnitude while scaling down the value of a and b. As a result, the effect of HC-type interfaces will be non-negligible with small-scale thermal devices.

## 3. Theoretical Analysis

The design of a thermal cloak in the transient regime, distinct from that in the steady regime, relates not only to the geometric parameters and conductivities but also to the volumetric heat capacity in each region. However, based on that we have assumed a constant volumetric heat capacity $s_{0}$ in both Region I and Region III, the volumetric heat capacity in Region II have to be identical to the neighboring regions such that the monopole constraint for thermal invisibility can be fulfilled in the quasi-static limit, irrespective of the bonding conditions. The dipole mode invisibility condition will prevail while the temporal frequency ω≈0, thereby, governing the long-term response as time elapses. Different from those for steady-state regimes, the thermal localization in Region I is difficult to be precisely manipulated at every instant of time for a transient response. However, within the quasi-static limit, we find that the manipulation of thermal localization in Region I is the same as that in the steady-state regime [27], still mainly controlled by the anisotropy ratio $\lambda =\sqrt{k_{\theta }/k_{r}}$. The perturbation of the controlled heat flux due to the transient response in the target region will eventually goes out with time. The value of volumetric heat capacity will affect the amount of perturbation over the transient response, and govern how long it will take for a steady state established.

When the value of $k_{\theta }$, or more precisely the value of λ, is greater than a critical value $\lambda_{cr}$, the heat flow will tend to be guided around Region II circumferentially. This will impede heat flux from entering Region I, exhibiting a shielding effect. Concentrating effect is opposite to the shielding behavior. When the radial conductivity $k_{r}$ in Region II is increased to a level such that $\lambda <\lambda_{cr}$, the heat flux will be directed toward the core region, leading to a concentrating effect in Region I. A value of $\lambda =\lambda_{cr}$ indicates the case without thermal localization in Region I. As mentioned in [27], the value of $\lambda_{cr}$ is highly pertinent to the bonding conditions at the interfaces. In the case of ideal interfaces, one has $\lambda_{cr}=1$, indicating that the thermal shielding (λ>1) or concentrating effect (λ<1) can be simply achieved by the value of λ. When there is imperfect bonding at the interfaces, the value of $\lambda_{cr}$ will be reduced in magnitude. This gives $\lambda_{cr}<1$ for either LC- or HC-type interfaces, as illustrated in [27]. This phenomenon physically explains by the fact that there shall be some of the heat energy dissipated while heat flow passes across the imperfect interface. As such, a shielding effect will be much more achievable than a concentrating effect in the presence of imperfect interfaces. In addition, one can find that the amplification ratio of concentrating effect is always bounded by the geometric parameter b/a as the steady state is nearly established, while a perfect shielding effect can be always achievable regardless of the bonding conditions at interfaces.

In Table 1 we list the weak invisibility conditions for a transient-state response in comparison with the strong invisibility conditions for a steady-state response [27]. Obviously, it can be seen that the weak invisibility condition in regard to the dipole mode for a transient response is identical to the strong invisibility condition for the steady-state case. It is of interest to mention that, mathematically the eigenfunctions of the dipole mode (n=1) in (3) will asymptotically approach the polynomials composed of $r^{\pm \lambda }$, which correspond to the eigenfunctions in the steady-state regime. This also offers an interpretation, explaining why the dipole mode constraint in the transient regime will be identical to the thermal invisibility condition in a steady state.

## 4. Methods

In addition to a homogeneous volumetric heat capacity, namely $s_{2}=s_{0}$, the design of a thermal cloak in the transient regime under the quasi-static limit can be constructed via a strategy as that proposed for the steady-state response [27]. According to the design methods, the design of a thermal cloak can be constructed via the concept of neutral inclusions [30,43] through a series of homogenization procedures based on a composite cylindrical assemblage (CCA) [44] model. Incorporating the effect of an imperfect interface, this method provides a systematic way to decompose the derivations into three separate steps, greatly simplifying the algebraic complexity. The first step will involve the homogenization of Region I coated with an imperfect interface at r=a. Secondly, the homogenization for the composite cylinder of the coated core region enclosed by the designed metamaterial, Region II, is determined. To optimally achieve invisibility, the effective material properties of the entire inclusion that concurrently concern the outer imperfect effect at r=b will be equivalent to that of the background medium under weak invisibility conditions. It is notable that only the second step is required for the cases of ideal interfaces. In Section 5, the numerical simulations based on the finite element method will be illustrated to validate our theoretical methodology.

This idea can be extended to design a multi-layered thermal metamaterial via a series of homogenization by applying two fundamental techniques recursively [27]. This can be utilized to realize a suitably designed thermal metamaterial with tailored orthotropic material properties and offers an excellent alternative in comparison with the classical method [45]. For the realization of a thermal metamaterial, the classical method suggests that a metamaterial with cylindrically anisotropic conductivities $k_{r}=2{\left(\kappa_{A}^{-1}+\kappa_{B}^{-1}\right)}^{-1}$ and $k_{\theta }=\left(k_{A}+k_{B}\right)/2$ can be fabricated as a layered heterogeneous composite material consisting of two alternating materials with isotropic conductivities $k_{A}$ and $k_{B}$ [45]. Nevertheless, this asymptotic approach requires a sufficient number of layers to render the cloak asymptotically equivalent to that made of a homogeneous material with cylindrically orthotropic conductivities $k_{r}$ and $k_{\theta }$, especially when the values of $k_{A}$ and $k_{B}$ differ greatly. In contrast, it is of our interest that, for the design of thermal cloaks via the concept of neutral inclusions, the thermal invisibility conditions not only can be always fulfilled irrespective of the number of annular layers but also be capable of counting into the effect of bonding imperfections at each interface.

## 5. Numerical Simulations

**Figure A2:**
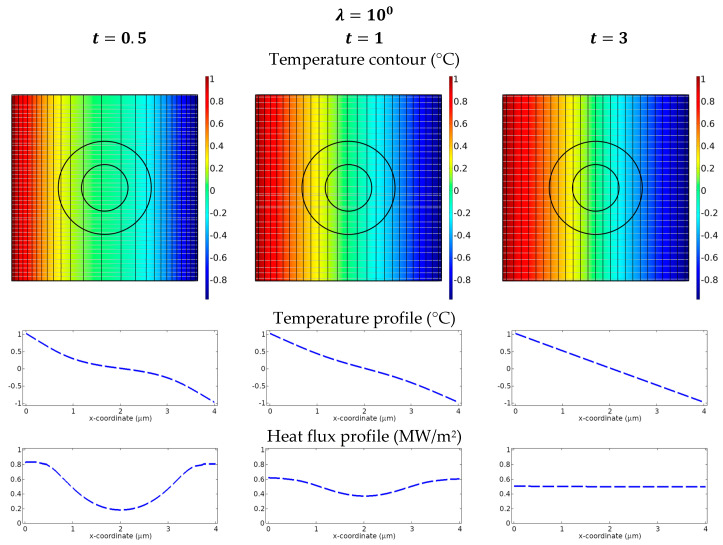
Temperature contours, together with heat flux profiles based on finite element simulations (COMSOL) for a background medium. Note that the configuration of Figure A2 is a homogeneous medium throughout which is taken as the reference medium in comparison with the designed cases. For numerical simulations, we consider b=1 μm and c=1/4. The temporal frequency is $\omega =10^{-4}$. The temperature profile as well as the heat flux profile along the x-axis are shown at the bottom. As a reference case, all the profile diagrams are illustrated with dashed lines to be compared with others. The temperature is described as Celsius (℃), while the heat flux is in MW/m^2^.

The thermal cloak based on the universal connections (10) and (11) for LC-type interfaces, and (15) and (16) for HC-type interfaces will be demonstrated to exhibit optimal cloaks that can suppress the disturbance in Region III to the maximum extent. In Figure A2 the reference case in which the whole medium is a homogeneous material is illustrated. To meet the requirement for the quasi-static assumption, a relatively small angular frequency of the incident excitation is taken as $\omega =10^{-4}$. Additionally, it is mentioned that the requirement of the quasi-static approximation, which is confined by $K_{0}b=\sqrt{i\omega s_{0}/k_{0}}b\ll 1$, can be guaranteed with a small size of the radius b, low value of $s_{0}$, or large $k_{0}$ as well. When the size of the thermal device is much smaller than the diffusion length, the scattered field will be very small and decay with time quickly. Thus a relatively small thermal device with a radius a=0.5 μm and area fraction c=1/4 is considered. On the other hand, a value of small volumetric heat capacity physically will barely store energy with time and converge to a steady state in a very short time. For numerical demonstration, a small value of $s_{0}=1$ MJ⋅K^−1^⋅m^−3^ is specified with appropriate thermal conductivity of $k_{0}=1$ W⋅K^−1^⋅m^−1^. Note that the volumetric heat capacities of natural solid materials vary roughly from about unity to 4 MJ⋅K^−1^⋅m^−3^ [46], for example, lead at 1.44 MJ⋅K^−1^⋅m^−3^, and iron at 3.569 MJ⋅K^−1^⋅m^−3^. The value of thermal conductivity of natural solids varies a great deal ranging from 0.02 W⋅K^−1^⋅m^−1^ (aerogel, recognized currently as the solid material with the lowest thermal conductivity) to about 2000 W⋅K^−1^⋅m^−1^ (for example diamond).

**Figure 2:**
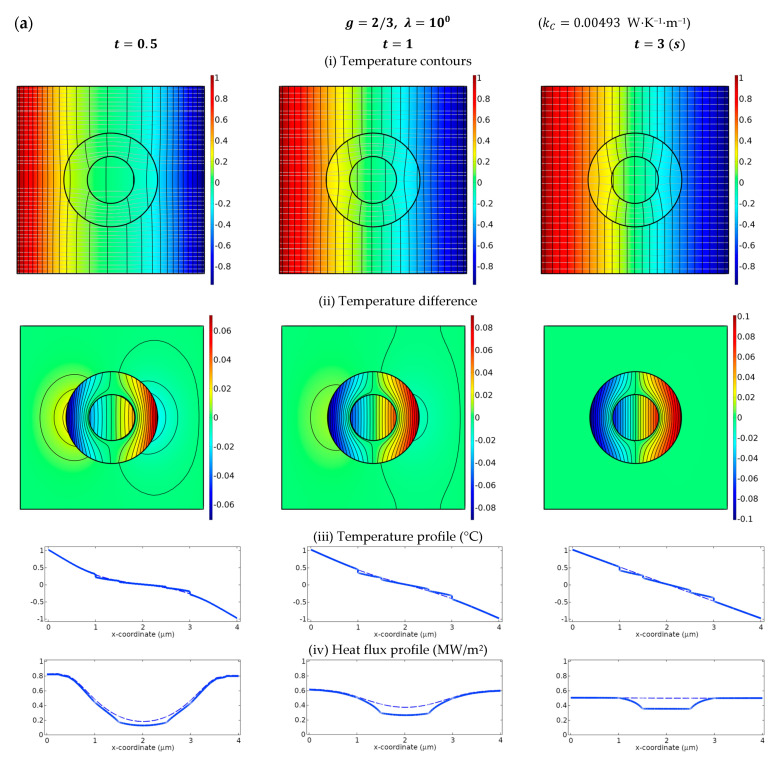

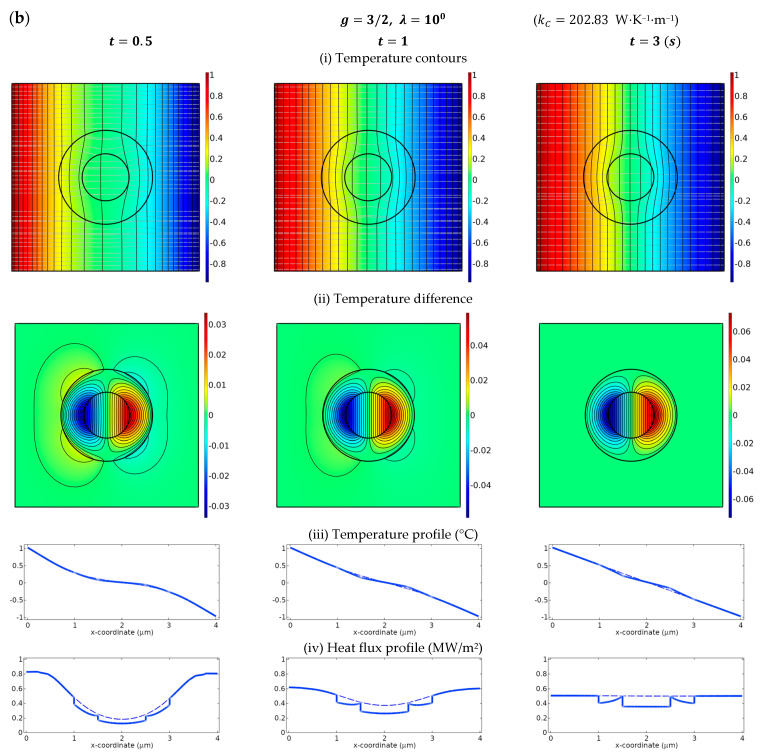
Temperature contours, together with temperature and heat flux profiles based on finite element simulations (COMSOL) for isotropic Region II with LC- and HC-type interfaces. The imperfectness parameter is specified as g=2/3 in (**a**) for LC-type, and g=3/2 in (**b**) for HC-type interfaces. The temporal frequency is $\omega =10^{-4}$. For numerical simulations, we consider b=1 μm and c=1/4. We plot the contour of the temperature difference between the concerned cases and the reference case illustrated in Figure A2. The temperature contours are shown to demonstrate the temperature field for the transient responses for various time periods in (i). The temperature difference between our concerned cases and Figure A2 are plotted in (ii) to highlight the scattering field in Region III. The temperature profile as well as the heat flux profile along the x-axis are shown in (iii) and (iv). The dashed lines in the profile figures are the results in Figure A2. Note that the value of $k_{C}$ (in W⋅K^−1^⋅m^−1^) is listed in each case for reference. The temperature is described in Celsius scale (°C), while the heat flux is in MW/m^2^.

In Figure 2, the case of $\lambda =10^{0}$ is considered which refers to an isotropic Region II. For numerical simulations, the imperfect interface can be simulated as an ultrathin interphase layer with thickness h and isotropic conductivity $k_{C}$ since the interfaces between two connected materials are always considered perfectly bonded in the finite element analysis software COMSOL. The value of $k_{C}$ is related to the imperfect interface parameters β and α in such a way that $k_{C}=\beta h$ [39] and $k_{C}=\alpha /h$ [29] for the LC- and HC-type interfaces respectively. As a result, we consider two interphase layers ranging from a to a+h and b to b+h respectively. For the cases of LC-type interfaces, the value of $k_{C}$ shall be extremely small compared with the conductivities of neighboring phase materials; while in contrast, $k_{C}$ will reach a value much higher than that of the adjacent contact materials for HC-type interfaces. Theoretically $k_{C}$ will approach zero or goes to unbounded respectively for the cases of LC- or HC-type interfaces while the thickness h→0. Here a sufficiently small value of $h=10^{-3}b=10^{-3}\mu m$ is employed to give a convergent solution. The corresponding value of $k_{C}$ for each case is listed in Figure 2. The parameter that signifies the bonding situations is specified as g=2/3 in Figure 2a and g=3/2 in (b) for the cases of LC- and HC-type interfaces respectively. The transient excitation $T_{inc}=U_{inc}e^{-i\omega t}$ is applied on the left-hand side, and $-T_{inc}$ on the right-hand side to possess the symmetric condition. In the first row in Figure 2, the temperature contours are plotted to demonstrate the temperature field for the transient responses at various periods of time. According to the concept of SCT, the perturbation in Region III shall be minimized while the weak invisibility conditions are fulfilled. The temperature difference between our concerned cases and Figure A2 below the temperature contours, denoted as (ii), are plotted to highlight the scattering field in Region III and demonstrate the shielding effect or concentrating effect in the core region. The temperature and heat flux profiles along x-axis are illustrated respectively in (iii) and (iv) below the contours to highlight the thermal localization as well as the jump across the interfaces on temperature or heat flux for LC- or HC-type cases respectively. The dashed lines in the profile diagrams represent the results in Figure A2. When the profiles of the studied case (solid lines) overlap with the referenced case (dashed lines) in Region III, the anisotropic region will be invisible to the background medium. From the results presented in [27], one can observe that an isotropic metamaterial in Region II with imperfect interfaces shall exhibit a shielding effect since $\lambda_{cr}<1$ for either type of imperfect interface.

It can be seen from the profile diagram in Figure 2a,b that the heat flux in Region I is reduced in amount compared with the dashed lines, exhibiting a shielding effect as it should be. On the other hand, the dashed lines in the temperature and flux profiles are nearly overlapped with the solid lines in Region III, resulting in excellent invisibility over time. As time elapses, the temperature difference in Region III will decrease. A steady-state response has been asymptotically established at the time t=6 s, at which the thermal cloak is almost invisible, and thus the temperature, as well as heat flux in Region I and III, will be linearly distributed along the horizontal direction, as can be seen in the profile diagrams.

**Figure 3:**
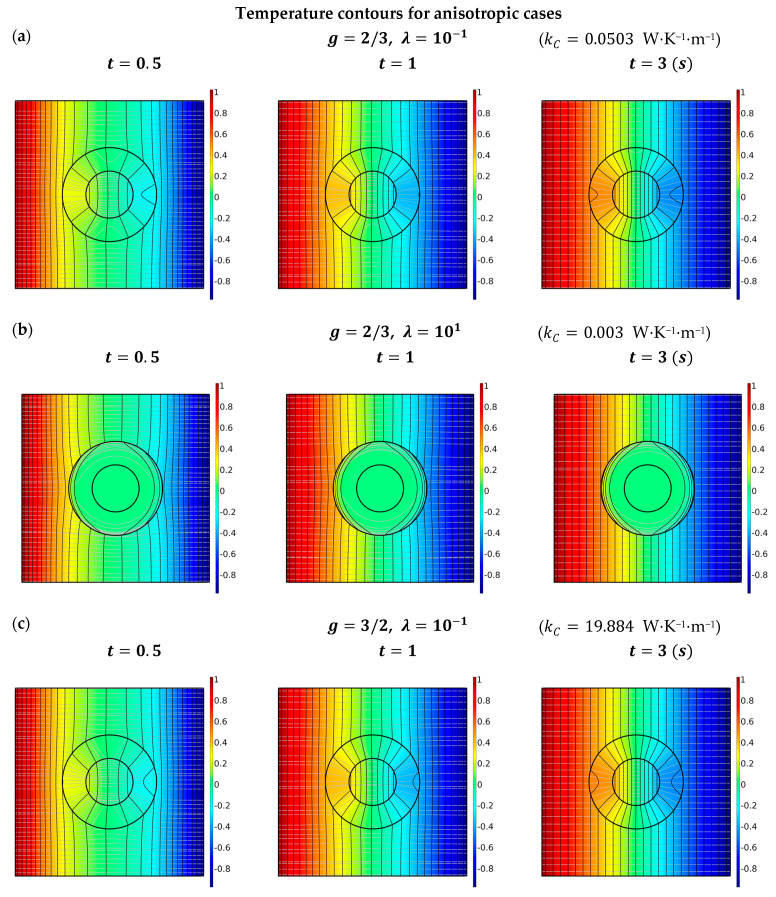

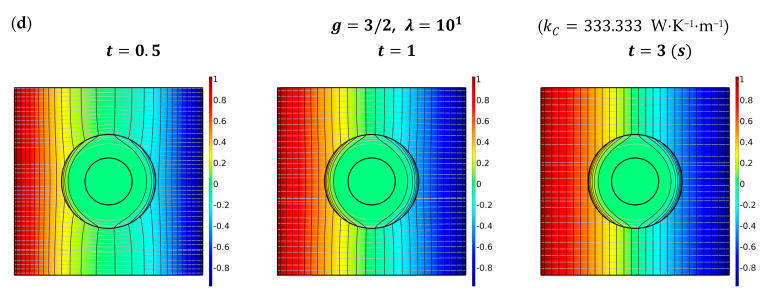
Temperature contours based on finite element simulations (COMSOL) for different values of λ with LC- and HC-type interfaces. The thermal localizations are presented in Region I for different anisotropy ratios in Region II. For numerical illustration, the imperfectness parameter g and anisotropy ratios λ are specified as (**a**) g=2/3 and $\lambda =10^{-1}$; (**b**) g=2/3 and $\lambda =10^{1}$; (**c**) g=3/2 and $\lambda =10^{-1}$; and (**d**) g=3/2 with $\lambda =10^{1}$. Note that the cases of g=2/3 will invoke the LC-type interfaces; whereas g=3/2 refers to the cases of HC-type interfaces. The geometric size and applied boundary conditions are identical to those described in Figure 2.

To demonstrate the thermal localization in Region I manipulated with the anisotropy ratio in Region II, the cases of $\lambda =10^{-1}$ and $\lambda =10^{1}$ are presented in Figure 3. In Figure 3a,c, the radial conductivity is one hundred times the circumferential conductivity, namely $k_{r}=10^{2}k_{\theta }$. As can be seen in the temperature contour diagram, the heat flux is directed toward the core region, and an evident concentrating effect is observed. In contrast, in Figure 3b,d we have $k_{\theta }=100k_{r}$ for the case of λ=10. The heat flux diffusing from Region III is guided around Region II, exhibiting an excellent shielding effect in Region I.

## 6. Conclusions

This work presents a detailed design of a thermal cloak with imperfect bonding interfaces focusing particularly on the transient state. It is shown analytically and demonstrated numerically that an object can only be made partially concealed under a transient-state condition with or without the effect of imperfect interfaces. The universal connections, also referred to as “weak invisibility conditions”, that can optimally suppress the visibility for the design of a thermal cloak made of homogeneous anisotropic material and constant volumetric heat capacity, were analytically found. It is remarkable to see that the weak invisibility conditions comprise two constraint conditions that correspond to two distinct modes, monopole and dipole modes. In the quasi-static limit, the monopole constraint condition, which confines the volumetric heat capacity of the metamaterial, characterizes the time effect on the invisibility condition. On the other hand, the dipole mode constraint condition, which only relates to the connections of conductivities, corresponds to the strong invisibility condition in the steady-state regime. This finding suggests that a thermal cloak designed via weak invisibility conditions will work excellently as an optimal solution that can minimize the visibility to the observers in the transient regime, and will turn into an ideal cloak whenever the steady state is achieved. Additionally, we also show that similar to those in the steady-state regime, the thermal localization in the quasi-static limit can be mainly controlled by the anisotropy of conductivities, and will be greatly influenced by the bonding conditions at the interfaces. A shielding effect is found to be always achievable. While the effect of concentrating will tend to slim down, especially when the imperfection arises at the interfaces. It is mentioned that the design concept could be applied to more complex geometric shapes, such as ellipses or ellipsoids. However, the mathematics involved will be much more complicated.

The present findings make new advances in theoretical fundamentals and numerical simulations on the effect of the imperfect interface in the transient regime and can serve as a guideline in the design of thermal metamaterials through the entire conduction process.

## Figures and Tables

**Table 1:** Thermal invisibility conditions for transient-state and steady-state situations.

	2D Steady State (Strong Invisibility Condition)	2D Transient State (Weak Invisibility Conditions)
Thermal invisibility conditions for perfect interfaces	$\sqrt{k_{\theta }k_{r}}=k_{0}$. [37]	$\sqrt{k_{\theta }k_{r}}=k_{0}$, for $S_{1}=0$ $s_{2}=s_{0}$. for $S_{0}=0$
Thermal invisibility conditions for LC-type interfaces	$g\frac{\left(g+\left(1+{\tilde{\beta }}_{a}\right)\right)-c^{\lambda }\left(g-\left(1+{\tilde{\beta }}_{a}\right)\right)}{\left(g+\left(1+{\tilde{\beta }}_{a}\right)\right)+c^{\lambda }\left(g-\left(1+{\tilde{\beta }}_{a}\right)\right)}+{\tilde{\beta }}_{b}=1$. [27]	$g\frac{\left(g+\left(1+{\tilde{\beta }}_{a}\right)\right)-c^{\lambda }\left(g-\left(1+{\tilde{\beta }}_{a}\right)\right)}{\left(g+\left(1+{\tilde{\beta }}_{a}\right)\right)+c^{\lambda }\left(g-\left(1+{\tilde{\beta }}_{a}\right)\right)}+{\tilde{\beta }}_{b}=1$, for $S_{1}=0$ $s_{2}=s_{0}$. for $S_{0}=0$
Thermal invisibility conditions for HC-type interfaces	$g^{-1}\frac{\left(g\left(1+{\hat{\alpha }}_{a}\right)+1\right)+c^{\lambda }\left(g\left(1+{\hat{\alpha }}_{a}\right)-1\right)}{\left(g\left(1+{\hat{\alpha }}_{a}\right)+1\right)-c^{\lambda }\left(g\left(1+{\hat{\alpha }}_{a}\right)-1\right)}+{\hat{\alpha }}_{b}=1$. [27]	$g^{-1}\frac{\left(g\left(1+{\hat{\alpha }}_{a}\right)+1\right)+c^{\lambda }\left(g\left(1+{\hat{\alpha }}_{a}\right)-1\right)}{\left(g\left(1+{\hat{\alpha }}_{a}\right)+1\right)-c^{\lambda }\left(g\left(1+{\hat{\alpha }}_{a}\right)-1\right)}+{\hat{\alpha }}_{b}=1$, for $S_{1}=0$ $s_{2}=s_{0}$. for $S_{0}=0$,

**Figure A1:**
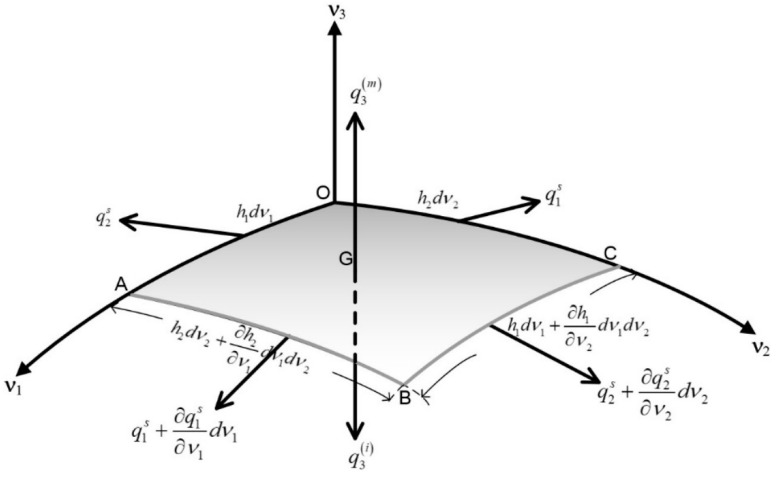
A schematic illustration of a small element on the curved interface subjected to the heat flux on the top face which is in contact with the matrix (denoted by the superscript m), on the bottom face which is in contact with the inclusion (denoted by the superscript i), and along the four edges (with superscript s).

## Data Availability

Data are contained within the article.

## Appendix A. Scattering Width for a Two-Dimensional System

In this Appendix A, we provide a detailed derivation for Equation (5). The scattering width $\sigma_{2D}$, defined in (4), is utilized to measure the visibility of the scattered field away from the inclusion [35]. Under the quasi-static approximation, with the substitution of $T\left(r,\theta ,t\right)=U\left(r,\theta \right)e^{-i\omega t}$ and (3)_3_ into (4), the scattering width can be expanded as

(A1) $$\begin{array}{ll} \sigma_{2D} & =\underset{r\to \infty }{\lim}\int \frac{{\left|T_{sc}\right|}^{2}}{{\left|T_{inc}\right|}^{2}}d\Omega \\ & =\underset{r\to \infty }{\lim}\int \frac{{\left|U_{sc}\right|}^{2}}{{\left|U_{inc}\right|}^{2}}d\Omega \\ & =\underset{r\to \infty }{\lim}\int \sum_{n=-\infty }^{\infty }\frac{{\left|i^{n}S_{n}H_{n}^{\left(1\right)}\left(K_{0}r\right)e^{in\theta }\right|}^{2}}{{\left|e^{iK_{0}r\cos\theta }\right|}^{2}}d\Omega \\ & =\underset{r\to \infty }{\lim}\int \sum_{n=-\infty }^{\infty }\frac{{\left|S_{n}H_{n}^{\left(1\right)}\left(K_{0}r\right)\right|}^{2}}{{\left|e^{iK_{0}r\cos\theta }\right|}^{2}}d\Omega . \end{array}$$

As dΩ=rdθ, the integration with respect to θ simply yields the value of 2π. As such, the value of $\sigma_{2D}$ can be simplified as

(A2) $$\sigma_{2D}=\underset{r\to \infty }{\lim}\left(2\pi r\sum_{n=-\infty }^{\infty }\frac{{\left|S_{n}H_{n}^{\left(1\right)}\left(K_{0}r\right)\right|}^{2}}{{\left|e^{iK_{0}r\cos\theta }\right|}^{2}}\right).$$

With consideration of the asymptotic expansion for Hankel function of the first kind with large argument [34]

(A3) $$H_{\nu }^{\left(1\right)}\left(x\right)\approx \sqrt{\frac{2}{\pi x}}e^{i\left(x-\frac{\nu \pi }{2}-\frac{\pi }{4}\right)},\;\mathrm{for}\;\nu \neq 0\;\mathrm{and}\;\mathrm{large}\;x,$$

the scattering width in (A2) can be expressed as

(A4) $$\begin{array}{ll} \sigma_{2D} & =\underset{r\to \infty }{\lim}\left(2\pi r\sum_{n=-\infty }^{\infty }\frac{{\left|\frac{S_{n}}{\sqrt{\pi r}}2^{\frac{1}{4}}{\left(K_{0}^{\prime }\right)}^{-\frac{1}{2}}e^{-K_{0}^{\prime }r}e^{i\left(K_{0}^{\prime }r-\frac{n\pi }{2}-\frac{3\pi }{8}\right)}\right|}^{2}}{{\left|e^{iK_{0}^{\prime }\left(1+i\right)r\cos\theta }\right|}^{2}}\right)\\ & =\underset{r\to \infty }{\lim}\left(2\pi r\sum_{n=-\infty }^{\infty }\frac{{\left|\frac{S_{n}}{\sqrt{\pi r}}2^{\frac{1}{4}}{\left(K_{0}^{\prime }\right)}^{-\frac{1}{2}}e^{-K_{0}^{\prime }r}\right|}^{2}}{{\left|e^{-K_{0}^{\prime }r\cos\theta }\right|}^{2}}\right), \end{array}$$

in which $K_{0}^{\prime }=\sqrt{\omega s_{0}/\left(2k_{0}\right)}$ is the real part of the wave number $K_{0}$, such that $K_{0}=\left(1+i\right)K_{0}^{\prime }$ as introduced in our main text. In the quasi-static limit, the value of $K_{0}^{\prime }$ is relatively small, thereby $e^{-K_{0}^{\prime }r}\to 1$. Consequently, we have

(A5) $$\sigma_{2D}=\underset{r\to \infty }{\lim}\left(\frac{4}{\left|K_{0}\right|}\sum_{n=-\infty }^{\infty }{\left|S_{n}\right|}^{2}\right).$$

Note that $\left|K_{0}\right|=\sqrt{2}K_{0}^{\prime }$ in (A5). Since the values of $S_{n}$ are independent of the radial coordinate r, the formula for determining the scattering width can be reduced to (5).

## Appendix B. Derivation of Weak Thermal Invisibility Conditions

In this Appendix B, we provide a derivation for the exact connections of thermal invisibility in the transient regime with imperfect interface effects. Specifically, a detailed derivation for the universal connections for thermal invisibility (10), (11), (15), and (16) in the transient regime under the quasi-static limit assumption will be given in detail. In cylindrical coordinates, the heat conduction equation $\nabla \cdot k\nabla T=s\left(\partial T/\partial t\right)$ can be written as

(A6) $$\frac{\partial^{2}T}{\partial r^{2}}+\frac{1}{r}\frac{\partial T}{\partial r}+\frac{k_{\theta }}{k_{r}}\frac{1}{r^{2}}\frac{\partial^{2}T}{\partial \theta^{2}}-\frac{s}{k_{r}}\frac{\partial T}{\partial t}=0.$$

With the assumption of $T\left(r,\theta ,t\right)=U\left(r,\theta \right)e^{-i\omega t}$, (A.6) can be rewritten as

(A7) $$\frac{\partial^{2}U}{\partial r^{2}}+\frac{1}{r}\frac{\partial U}{\partial r}+\frac{k_{\theta }}{k_{r}}\frac{1}{r^{2}}\frac{\partial^{2}U}{\partial \theta^{2}}+K^{2}U=0,$$

where $K=\sqrt{i\omega s/k_{r}}$ refers to the wave number, $k_{\theta }$ and $k_{r}$ denote the circumferential conductivity and radial conductivity respectively. Note that for Region I and Region III, $k_{\theta }=k_{r}=k_{0}$. In Region II the anisotropy parameter is denoted as $\lambda =\sqrt{k_{\theta }/k_{r}}$. With the variable decomposition of $U\left(r,\theta \right)=U\left(r\right)e^{in\theta }$, (A7) will become a Bessel differential equation whose solutions consist of two series of independent Bessel functions. As such, the scalar temperature fields that fulfill Fourier’s relation can be expressed in cylindrical coordinates as described in (3).

### Appendix B.1. LC-Type Interfaces

For LC-type interfaces, the interface conditions (9) can be rewritten in matrix form with the substitution of (3) as

(A8) $$\tilde{A}x=\tilde{b},$$

where $x={\left(\begin{array}{cccc} A_{n} & B_{n} & C_{n} & S_{n} \end{array}\right)}^{T}$ is the undetermined coefficient vector. The matrix $\tilde{A}$ and vector $\tilde{b}$ are given as

(A9) A~=−JnK0a−k0βaK0Jn′K0aJλnK2aHλn1K2a0−k0K0Jn′K0akrK2Jλn′K2akrK2Hλn1′K2a00−JλnK2b−Hλn1K2binHn1K0b−k0βbK0inHn1′K0b0−krK2Jλn′K2b−krK2Hλn1′K2bk0K0inHn1′K0b,

and

(A10) b~=00−inJnK0b+k0βbK0inJn′K0b−k0K0inJn′K0b.

Note that the prime notation stands for the derivative with respect to the argument Kr. To our concern, the scattering coefficient $S_{n}$ can be derived from Cramer’s rule as $S_{n}={∆}_{S_{n}}/∆$, in which $∆=\det\left(\tilde{A}\right)$. The value of ${∆}_{S_{n}}$ is given by

(A11) ∆Sn=det−JnK1a−k0βaK0Jn′K0aJλnK2aHλn1K2a0−k0K0Jn′K0akrK2Jλn′K2akrK2Hλn1′K2a00−JλnK2b−Hλn1K2b−inJnK0b+k0βbK0inJn′K0b0−krK2Jλn′K2b−krK2Hλn1′K2b−k0K0inJn′K0b.

The boxed terms in (A9)–(A11) are marked to highlight the difference between the case of LC-type interfaces and that of perfect interfaces. It will recover back to the case of perfect interfaces when all the boxed terms vanish. Based on the concept of the scattering cancellation technique [31], we are aiming to eliminate the first two orders of scattering fields, namely $S_{0}$ and $S_{1}$. Equivalently, the weak invisibility conditions can be derived by letting ${∆}_{S_{0}}=0$ and ${∆}_{S_{1}}=0$ simultaneously. This will give the results shown in (10) and (11) in the main text with the quasi-static approximation.

### Appendix B.2. HC-Type Interfaces

With the substitution of the admissible temperature fields (3), the interface conditions for the HC-type cases (14) can be rewritten in matrix form

(A12) $$\hat{A}x=\hat{b}.$$

The matrix $\hat{A}$ and vector $\hat{b}$ are given as

(A13) A^=−JnK0aJλnK2aHλn1K2a0−k0K0Jn′K0a+iωss−αan2a2JnK0akrKIIJλn′K2akrKIIHλn1′K2a00−JλnK2b−Hλn1K2binHn1K0b0−krKIIJλn′K2b+iωss−αbn2b2JλnK2b−krKIIHλn1′K2b+iωss−αbn2b2Hλn1K2bk0K0inHn1′K0b

and

(A14) $$\hat{b}=\left(\begin{array}{c} 0\\ 0\\ -i^{n}J_{n}\left(K_{0}b\right)\\ -k_{0}K_{0}i^{n}J_{n}^{\prime }\left(K_{0}b\right) \end{array}\right).$$

Similarly, the scattering coefficient $S_{n}$ can be obtained by Cramer’s rule as $S_{n}={∆}_{S_{n}}/∆$, where $∆=\det\left(\hat{A}\right)$. The value of ${∆}_{S_{n}}$ is given by

(A15) ∆Sn=−JnK0aJλnK2aHλn1K2a0−k0K0Jn′K0a+iωss−αan2a2JnK0akrKIIJλn′K2akrK2Hλn1′K2a00−JλnK2b−Hλn1K2b−inJnK0b0−krKIIJλn′K2b+iωss−αbn2b2JλnK2b−krK2Hλn1′K2b+iωss−αbn2b2Hλn1K2b−k0K0inJn′K0b.

We mention that the boxed terms in (A13)–(A15) are those that mark the effect of the HC-type interfaces. The case will be reduced back to that of the perfect interfaces as introduced when the boxed terms are taken out. The host medium, Region III, can be made least perturbed by canceling out the first two modes of scattering field [31], namely $S_{0}$ and $S_{1}$. The weak invisibility conditions yielded by eliminating $S_{0}$ and $S_{1}$ can be obtained alternatively by letting ${∆}_{S_{0}}=0$ and ${∆}_{S_{1}}=0$. Consequently, the non-trivial results will lead to weak invisibility conditions for HC-type cases as described in (15).

## Appendix C. Derivation of the Interface Condition for Heat Flux with Surface Effect under Transient Regime

In this Appendix C, the interface condition for an HC-type interface that characterizes the discontinuity in heat flux due to the surface effect is constructed via the dynamic equilibrium balance along the curved surface. The procedure follows the geometric exposition of interface conditions with surface effect, originally proposed for surface elasticity [47]. Let us consider a curved surface between two bulk phases (Figure A1). Here for convenience, we refer to it as matrix and inclusion. The net heat energy per unit of time under the time-harmonic condition is proportional to the time-derivative of the temperature multiplied by the volumetric heat capacity $s=\rho c_{p}$, i.e., ∇·q=−s(∂T/∂t).

In reference to the exposition [47], the net heat energy across the four edges as well as the top and bottom faces are

(A16) $${\left.H\right|}_{OABC}=\left(q_{3}^{\left(m\right)}-q_{3}^{\left(i\right)}\right)h_{1}h_{2}d\nu_{1}d\nu_{2},\;{\left.H\right|}_{AB+OC}=\frac{\partial }{\partial \nu_{1}}\left(q_{1}^{s}h_{2}\right)d\nu_{1}d\nu_{2},\;{\left.H\right|}_{OA+BC}=\frac{\partial }{\partial \nu_{2}}\left(q_{2}^{s}h_{1}\right)d\nu_{1}d\nu_{2}.$$

The superscript (m) and (i) refer to the matrix and inclusion respectively, whereas the superscript s denotes the surface quantities. Hence the balance equation becomes

(A17) $$q_{3}^{\left(m\right)}-q_{3}^{\left(i\right)}+\frac{1}{h_{1}h_{2}}\left(\frac{\partial }{\partial \nu_{1}}\left(q_{1}^{s}h_{2}\right)+\frac{\partial }{\partial \nu_{2}}\left(q_{2}^{s}h_{1}\right)\right)=-s_{s}\frac{\partial T}{\partial t},$$

where $s_{s}=s_{int}h$ refers to the surface volumetric heat capacity, which is defined as the volumetric heat capacity of the interphase layer $s_{int}$ multiplied by the thickness of the interface h. The surface heat flux $q_{\gamma }^{s}$ (γ=1,2) is the sum of the heat flux of the interphase layer along the thickness. Substitution of q=−k∇T into the above equation gives

(A18) $$-\frac{1}{h_{3}}\left(k_{3}^{\left(m\right)}\frac{\partial T^{\left(m\right)}}{\partial \nu_{3}}-k_{3}^{\left(i\right)}\frac{\partial T^{\left(m\right)}}{\partial \nu_{3}}\right)+\frac{k_{C}h}{h_{1}h_{2}}\left(\frac{\partial }{\partial \nu_{1}}\left(\frac{h_{2}}{h_{1}}\frac{\partial T}{\partial \nu_{1}}\right)+\frac{\partial }{\partial \nu_{2}}\left(\frac{h_{1}}{h_{2}}\frac{\partial T}{\partial \nu_{2}}\right)\right)=-s_{s}\frac{\partial T}{\partial t}.$$

The interface can be viewed as an interphase layer with a very small thickness h and with isotropic conductivity $k_{C}$, the parameter $\alpha =\underset{h\to 0,k_{C}\to \infty }{\lim}k_{C}h$ representing the effect of the high-conductive interface. Thus, the interface condition (A18) expressed in cylindrical coordinates turns into the form of

(A19) $${\left.\left(k_{r}^{\left(m\right)}\frac{\partial T^{\left(m\right)}}{\partial n}-k_{r}^{\left(i\right)}\frac{\partial T^{\left(i\right)}}{\partial n}\right)\right|}_{\Gamma }=\alpha {∆}_{s}T+s_{s}\frac{\partial T}{\partial t},$$

where ${∆}_{s}$ stands for the surface Laplacian operator, and n indicates the normal direction. Equation (A19) characterizes the surface effect that the interface between the matrix and inclusion undergoes a heat flux jump with a time effect.

## Appendix D. Numerical Simulations of a Homogeneous Background Medium

In Figure A2, we illustrate the numerical results for the reference case in which the whole medium is homogeneously made of a material with isotropic conductivity $k_{0}$ and constant volumetric heat capacity $s_{0}$. Hence in this case Region III 2 will remain thoroughly undisturbed during the process of conduction. The geometric configuration is the same as that in Figure 2. Temperature and flux profiles are illustrated below the contours.
